# Predictors of uptake of eye examination in people living with diabetes mellitus in three counties of Kenya

**DOI:** 10.1186/s41182-017-0080-7

**Published:** 2017-12-21

**Authors:** Nyawira Mwangi, David Macleod, Stephen Gichuhi, Lawrence Muthami, Consuela Moorman, Covadonga Bascaran, Allen Foster

**Affiliations:** 1 London School of Hygiene and Tropical Medicine, London, UK; 2 Kenya Medical Training College, Nairobi, Kenya; 3 University of Nairobi, Nairobi, Kenya; 4 Kenya Medical Research Institute, Nairobi, Kenya; 5 Oxford University Hospitals NHS Trust, Oxford, UK

**Keywords:** Diabetes, Diabetic retinopathy, Eye examination, Access, Screening, Kenya, Sub-Saharan Africa

## Abstract

**Background:**

Diabetic retinopathy (DR) is a significant public health concern that is potentially blinding. Clinical practice guidelines recommend annual eye examination of patients with diabetes for early detection of DR. Our aim was to identify the demand-side factors that influence uptake of eye examination among patients already utilizing diabetes services in three counties of Kenya.

**Methods:**

We designed a clinic based cross-sectional study and used three-stage sampling to select three counties, nine diabetes clinics in these counties and 270 patients with diabetes attending these clinics. We interviewed the participants using a structured questionnaire. The two outcomes of interest were ‘eye examination in the last 12 months’ and ‘eye examination ever’. The exposure variables were the characteristics of participants living with diabetes.

**Results:**

The participants had a mean age of 53.3 years (SD 14.1) and an average interval of 4 months between visits to the diabetes clinic. Only 25.6% of participants had ever had an eye examination in their lifetime, while 13.3% had it in the preceding year. The independent predictors of uptake were referral by diabetes services, patient knowledge of diabetes eye complications, comorbid hypertension and urban or semi-urban residence.

**Conclusions:**

We conclude that access to retinal examination for DR is low in all three counties. An intervention that increases the knowledge of patients with diabetes about eye complications and promotes referral of patients with diabetes for eye examination may improve access to annual eye examination for DR.

## Background

Diabetes mellitus (DM) causes visual impairment and blindness through diabetic eye disease, which includes cataract and diabetic retinopathy. Diabetic retinopathy (DR) is a progressive microvascular complication of diabetes. Approximately one third (34.6%) of people living with diabetes (PLWD) have DR and 10% have sight-threatening DR (STDR) [1]. The increasing magnitude of DM and DR is a significant public health challenge [2, 3]. There is strong evidence for the cost-effectiveness of screening for DR in prevention of blindness [4, 5].

There are several reasons why access to eye examination for PLWD in Kenya is important. First, the prevalence and magnitude of DM and DR is increasing. An estimated 14.2 million people in the African region had diabetes in 2015 [6]. This number is expected to increase by 140% between 2015 and 2040 [6]. The greatest increase is predicted to be in countries transitioning from low to middle income, like Kenya. The prevalence of diabetes in the Kenyan population aged 20–79 years was 2.2% in 2015 [7]. This translates into 484,000 adults with diabetes, of whom approximately one third have DR (150,000–170,000) and 10% (40,000–50,000) have STDR. Second, both DR and STDR are asymptomatic and STDR can progress to blindness if not treated early [8–10]. An eye examination of the retina through a dilated pupil, usually annual, can identify those with DR who are at risk of developing STDR and needing treatment [10, 11]. Third, treatment of patients who have STDR reduces the risk of vision loss [12–14].

The determinants of access to retinal examination are complex and include both supply and demand factors [9, 15]. Understanding the demand-side factors facilitates the development of targeted demand-side interventions that reduce the barriers and support the enablers to increase the uptake of eye examination. Several studies have examined the use of eye care among patients with diabetes in America, Asia, Europe, and Oceania [16–21]. Many studies in Africa have focused on access to eye care for cataract but not DR [22–30]. In this paper, we report on factors influencing the uptake of eye examination for DR in PLWD. We define this test as a retinal examination through a dilated pupil conducted by an eye care worker using either an ophthalmoscope or retinal camera.

### Research in context panel

#### Evidence before this study

We searched Ovid MEDLINE, Cochrane Library, and EMBASE (2000–2016) using the terms ‘diabetes’ and ‘diabetic retinopathy’ in combination with the following terms: ‘access’, ‘screening’ and ‘eye examination’. We also searched cited references in articles identified by this search strategy. The evidence is that uptake of annual retinal examination is low in resource-poor settings (Table 1). However, the predictors of uptake of retinal examination have not been documented.

**Table 1:** Summary of other studies in developing countries

Study	Current study	Mumba et al. [32]	Onakpoya et al. [36]	Njambi, L [33]	Adriono et al. [19]	Wang et al. [20]	Shivashankar et al. [34]	GV Murthy et al. [35]
Country	Kenya	Tanzania	Nigeria	Kenya	Indoneshia	China	Delhi, India	11 cities, India
Year	2016	2009	2009	2012	2011	2010	2016	20
Target PLWD population	Adults in nine diabetes clinics	Adults in one diabetes clinic in a tertiary hospital	Adults in one diabetes clinic	Adults attending a diabetes clinic in one hospital	Adults in three clinics	Adults attending health facilities	Adults attending 23 primary care clinic	Adults attending diabetes hospitals/clinics
Sample size	270	316	84	253	196	824	406	285
Screening rate (last 12 months)	13.3%	28%	Not reported	Not reported	15.3%	33.3%	7.4%	Not reported
Screening rate (ever)	25.6%	59.1%	28.9%	29%	Not reported	56.8%	Not reported	67.7%

#### Added value of this study

We found the uptake of retinal examination among patients utilising diabetes services in three counties of Kenya to be even lower than documented in other studies. Predictors of uptake of retinal examination were (a) referral from diabetes services, (b) knowledge of diabetes eye complications and (c) comorbid hypertension. About half of the patients had the perception that a retinal examination was not necessary in the absence of ocular symptoms. Using this evidence, we present a conceptual model on how to improve uptake of retinal examination.

#### Implications of all the available evidence

An intervention to reverse low uptake of retinal examination should include both health education and referral pathway interventions. From our findings, the education component should prioritize two aspects of knowledge: (1) information on diabetes eye complications and (2) information on eye examinations (importance and frequency). The referral intervention should address barriers to uptake of examination. These interventions are potentially cost-effective and may also strengthen integration of diabetic retinopathy screening into diabetes services.

This study was conducted when Kenya has just completed a STEPwise survey on risk factors for non-communicable diseases and determined the prevalence of DM. It could form the baseline from which trends in uptake of retinal examination can be compared as prevalence of DM increases in the next decade.

## Methods

### Study setting

This study was part of a cross-sectional health system assessment for diabetes and diabetic retinopathy. A three-stage sampling process was used. Three counties were purposively selected to represent different environments and populations within the diabetes belt in Kenya: Kirinyaga (predominantly rural), Nakuru (semi-urban) and Nairobi (urban). Three diabetes outpatient clinics were selected in each county. A list of public, private and faith-based clinics in each country was obtained, and 1 clinic was selected in each category through random sampling. In each of these nine diabetes clinics, 30 patients were selected by random sampling from the PLWD attending the clinic on the day of interview. The list of male and female patients was used as the sampling frame, with a random starting point and a regular sampling interval of between three and five depending on the volume of the patients attending each clinic. This procedure made it possible to recruit an equal number of men and women. A minimum sample size of 73 per county (thereafter increased to 90) was determined based on the estimate that 5% of the population of PLWD attending diabetes clinics have an annual dilated eye examination, with the desirable degree of accuracy set at 0.05.

The study followed the tenets of the World Medical Association’s Declaration of Helsinki. The London School of Hygiene and Tropical Medicine and African Medical Research Foundation granted ethical approval. All participants gave written informed consent.

### Participants

Eligible persons included those 18 years of age or older, known to have diabetes, resident in the county, receiving services at participating outpatient diabetes clinics, and willing to participate in the study. Non-residents in the county and acutely ill patients were excluded.

The primary investigator and research assistants interviewed the participants in English or Kiswahili using a pretested structured questionnaire. Prior to data collection, the questionnaire was reviewed by local diabetologists, ophthalmologists and statisticians. A pilot test with diabetes patients was conducted in two different diabetes clinics within the study area (which were not part of the study sample).

Participation was voluntary and participants did not receive any financial incentives. The questionnaire had four broad categories of questions for PLWD: (a) sociodemographic characteristics, (b) experience with diabetes services, (c) knowledge of complications of diabetes and (d) experience with examination for complications of diabetes (including DR). All subjects reporting previous eye examinations were questioned as to whether the eye care worker instilled eye drops to dilate the pupils before the eye examination. This differentiated a regular eye examination and a dilated eye examination.

### Statistical analysis

STATA version 14 was used for data analysis [31]. The study had two outcomes of interest: ‘eye examination in the last 12 months’ and ‘eye examination ever’. Both were dichotomous ‘yes’ and ‘no’ variables. The exposure variables were characteristics of participants in relation to living with diabetes.

Descriptive statistics were shown as counts and percentages for categorical variables, and means and standard deviations for continuous variables. For each of the two outcomes of interest, tests of crude association were performed using chi-square tests for categorical exposure variables and *t* tests for continuous variables. Univariate logistic regression was used to identify exposure variables that were predictors of uptake of examination in the last year, and in analysing the ever had an eye exam outcome, all logistic regressions were adjusted for age. Multivariable analysis was performed using forward stepwise selection where exposure variables with the lowest *p* value were sequentially added to the regression model, using a cutoff for inclusion in the model of *p* < 0.05.

#### Role of funding source

The funders did not participate in study design, data collection, analysis, writing of the paper or submission for publication.

## Results

### Outcome variables: uptake of dilated eye examination

Ninety participants were interviewed in each county (*n* = 270). None of the participants declined to participate, and data for all variables was collected for all participants. Only 25.6% (*n* = 69) had ever had fundoscopy, while only 13.3% (*n* = 36) had been examined in the preceding year. The uptake of eye examination in other resource-poor settings is shown in Table 1.

### Exposure variables: participant characteristics

The mean age of participants was 52.3 years (SD 14.1, range 25–88 years). Approximately 47% were male, 23.7% had a family history of diabetes and 37.4% had comorbid hypertension. The mean duration of diabetes was 7.3 years (SD 5.5), and participants attend the diabetes clinic every 4 months (SD 1.5) on average. The main reason for that frequency is the physician’s recommendation. The other variables are shown on the first column of Table 2.

**Table 2:** Patient characteristics and association with eye examination

Variable	Summary of participants characteristics	Retinal exam last 12 months			Retinal exam ever		
	*N* (%) Mean (SD)	Had eye exam	No eye exam	*p* value	Had eye exam	No eye exam	*p* value
Number (%) in each category	270	36 (13.3%)	234 (86.3%)		69 (25.6)	201 (74.4)	
Number (%) by county				0.07			0.002
Kirinyaga	90	6 (6.7%)	84 (93.3%)		11 (12.2)	79 (87.8)	
Nairobi	89	14 (15.7%)	75 (84.3%)		29 (32.6)	60 (67.4)	
Nakuru	91	16(17.6%)	75 (82.4%)		29 (31.9)	62 (68.1)	
Age (mean years, SD)	53.3 (14.1)	57.1 (11.7)	52.7 (14.4)	0.08	60.5 (13.8)	50.8 (13.4)	< 0.0001
Sex (no. %)				0.7			0.5
Men	127 (47%)	18 (14.2)	109 (85.8)		35 (27.6)	92 (72.4)	
Women	144 (53%)	18 (12.5)	126 (87.5)				
Literacy				0.3			0.05
Primary or below	88 (32.8%)	13 (14.8)	75 (85.2)		30 (34.1)	58 (65.9)	
Secondary	111 (41.4%)	11 (9.9)	100 (90.1)		21 (18.9)	90 (81.1)	
Post-secondary	69 (25.8%)	12 (17.4	57 (82.6)		18 (26.1)	51 (73.9)	
Occupation				0.4			0.014
Unemployed	70 (25.9%)	6 (3.6)	64 (91.4)		19 (27.1)	51 (72.9)	
Low skilled	70 (25.9%)	9 (12.9)	61 (87.1)		14 (20)	56 (80)	
Professional	90 (33.3%)	13 (14.4)	77 (85.6)		18 (20)	72 (80)	
Retired	40 (33.3%)	8 (20)	32 (80)		18 (45)	22 (55)	
Duration of diabetes (mean years, SD)	7.3 (5.5)	8.9 (4.5)	7.1 (5.6)	0.06	9.4 (5.5)	6.6 (5.3)	0.0002
Interval of diabetes clinic visits (months)	4.0 (1.5)	4.3 (1.3)	4.0 (1.5)	0.4	4.3 (1.4)	3.9 (1.5)	0.08
Referred for eye examination				< 0.001			< 0.001
Yes	66 (24.4%)	23 (34.9)	43 (65)		47 (68.1)	19 (28.8)	
No	204 (75.6%)	13 (6.4)	191 (93.6)		22 (10.7))	182 (89.2)	
Perceived level of glucose control				0.02			0.4
Very good	10 (3.7%)	0	10 (100)		2 (20)	8 (80)	
Well	73 (27%)	17 (23.3)	56 (76.7)		23 (31.5)	50 (68.5)	
Adequate	107 (39.6%)	9 (8.4)	98 (91.6)		24 (22.4)	83 (77.6)	
Poor	68 (25.2%)	10 (14.7)	58 (85.3)		19 (27.9)	49 (72.1)	
Very poor	12 (4.4%)	0	12 (100)		1 (8.3)	11 (91.7)	
Diabetes in family member				0.8			0.6
Yes	64 (23.7%)	8 (12.5)	56 (87.5)		18 (28.1)	46 (71.9)	
No	206 (76.3%)	28 (13.6)	178 (86.4)				
Information on diabetes given at health facility				0.3			0.8
Yes	205 (75.9%)	30 (14.6)	175 (85.4)		53 (25.9)	152 (74.2)	
No	65 (24.1%)	6 (9.2)	59 (90.8)		16 (24.6)	49 (75.4)	
Knowledge of diabetes complications				0.4			0.9
Yes	103 (38.1%)	16 (15.5)	87 (84.5)		26 (25.2)	77 (74.8)	
No	167 (61.9%)	20 (12)	146 (88)		43 (25.8)	124 (74.3)	
Knowledge of diabetes eye complications				0.001			0.001
Yes	150 (55.6%)	29 (19.3)	121 (80.7)		50 (33.3)	100 (66.7)	
No	120 (44.4%)	7 (5.8)	113 (94.2)		19 (15.8)	101 (84.2)	
Comorbid hypertension				0.02			0.04
Yes	101 (37.4%)	20 (19.8)	81 (80.2)		33 (32.7)	68 (67.3)	
No	169 (62.6%)	16 (9.5)	153 (90.5)		36 (21.3)	133 (78.7)	
Opinion on need for an eye examination				*P* < 0.001			*P* < 0.001
No need	51 (18.9%)	1 (2.0)	50 (98)		6 (11.8)	45 (88.2)	
Only for ocular symptoms	115 (42.6%)	15 (13)	100 (87)		27 (23.5)	88 (76.5)	
Acceptable	80 (29.6%)	13 (16.3)	67 (83.8)		25 (28.8)	57 (71.3)	
Already doing it	9 (3.3%)	5 (55.6)	4 (44.4)		9 (100)	0	
Other opinion	15 (5.6%)	13 (13.3)	2 (86.7)		4 (26.7)	11 (73.3)	

### Determinants of eye examination

Table 2 also shows the patient-level determinants for fundoscopy. Only 24.4% had been referred from the diabetes clinic for a retinal examination, and 13.3% had taken this examination (fundoscopy**)** *in the last 12 months*. Variables that had the strongest evidence of an association with having had the exam in the last 12 months were (a) referral for an eye examination (*p* < 0.001), (b) knowledge of diabetes eye complications (*p* = 0.002), (c) comorbid hypertension (*p* = 0.02) and (d) county of residence (*p* = 0.07) (Table 3). Participants referred for an eye exam had almost eight times the odds of having attended an eye exam in the last 12 months compared to those who had not been referred (OR 7.9, 95% CI 3.7–16.4, *p* < 0.001). Participants who had a knowledge of diabetes eye complications had four times the odds (OR 3.9, CI 1.6–9.1) of attending as those who had no knowledge of eye complications. Hypertensive individuals had twice the odds of attendance, compared to those with normal blood pressure (OR 2.3, CI 1.1–4.7). The PLWD in Kirinyaga (rural) were the least likely to have had an eye examination in the last 12 months, with PLWD in Nairobi (urban) having 2.6 times the odds (CI 1.1–7.1) and PLWD in Nakuru (semi-urban) having three times the odds (CI 1.1–8.0).

**Table 3:** Predictors of eye examination last 12 months

Variable	Eye exam last 12 months		Eye exam ever	
	OR (95% CI)	*p* value	OR (95% CI)	*p* value
Demographic factors				
Increasing age (every year)	1.2 (1.1–1.6)	0.08	1.1 (1.0–1.1)	< 0.001
Male gender	1.1 (0.6–2.3)	0.7	1.2 (0.7–2.1)	0.5
County of residence (compared to Kirinyaga)				
Nakuru	3.0 (1.1–8.0)	0.03	3.4 (1.6–7.5)	0.02
Nairobi	2.6 (1.1–7.1)	0.06	3.5 (1.6–7.5)	0.02
Education				
Post-secondary education	1.1 (0.5–2.8)	0.8	0.7 (0.3–1.4)	0.3
Occupation (as compared to the unemployed)				
Professional	1.8 (0.6–5.0)	0.3	0.7(0.3–1.5)	0.3
Retired	2.7 (0.9–8.3)	0.09	2.2 (1.0–5.0)	0.06
Duration of diabetes	1.1 (1.0–1.1)	0.06	1.0 (1.0–1.1)	< 0.001
Referral for eye examination	7.9 (3.7–16.4)	< 0.001	20.5 (10.2–40.9)	< 0.001
Knowledge of diabetes complications	3.9 (1.6–9.2)	0.002	2.7 (1.5–4.8)	0.001
Comorbid hypertension	2.3 (1.1–4.7)	0.02	1.8 (1.0–3.1)	0.04

The main predictors for having *ever* had fundoscopy included (a) referral for eye examination (OR 20.5, CI 10.2–40.9, *p* < 0.001), (b) knowledge of diabetes eye complications (OR 2.7, CI 1.5–4.8, *p* < 0.001), (c) county (*p* = 0.02) and (d) comorbid hypertension (OR 1.8 CI 1.0–3.1 *p* = 0.02). The PLWD in Nakuru or Nairobi had three times the odds of attendance as compared in Kirinyaga (OR 3.4, CI 1.6–7.5 and OR 3.5, CI 1.6–7.5) (Table 3).

There was strong evidence of association of having a dilated eye examination (ever) with both increasing age and duration of diabetes (*p* < 0.0001), but the effect size was quite small, with the odds increasing by 1.1 times each year (thus, 2.6 times every decade), Table 3. In multivariable analysis, (a) referral, (b) knowledge of diabetes eye complications and (c) county of residence remained independent predictors for fundoscopy. Referral and knowledge of diabetes eye complications had the strongest relationship with uptake of eye examination and thus were included in the final multivariable analysis model. Interaction between referral and knowledge of diabetes eye complications was tested, and these remained significant independent predictors (*p* < 0.0001).

As referral for examination (ever) was the strongest predictor of uptake of examination, the variables associated with referral were analysed. The main exposure variables positively associated with referral (Table 4) were (a) increasing age (*p* < 0.0001), (b) longer duration of diabetes (*p* = 0.0005), (c) knowledge of diabetes eye complications (*p* = 0.003), (d) positive opinion on need for an eye examination (*p* < 0.001), (e) retirement (*p* = 0.01) and (f) residence in Nairobi or Nakuru (*p* = 0.03).

**Table 4:** Variables associated with referral for eye examination

Variable	Referred	Not referred	*p* value
Number (%) in each category	66 (24.4)	204 (75.6)	
Number (%) by county			0.03
Kirinyaga	15 (16.7)	75 (83.3)	
Nairobi	30 (33.8)	59 (66.3)	
Nakuru	21 (23.1)	70 (76.9)	
Age mean years, SD	59.8 (13.3)	51.2 (13.8)	< 0.0001
Sex *N* (%)			0.09
Male	37 (29.1)	90 (70.9)	
Female	29 (20.3)	114 (79.7)	
Occupation *N* (%)			0.01
Unemployed	17 (24.3)	53 (75.7)	
Low skilled	13 (18.6)	57 (81.4)	
Professional	18 (20)	72 (80)	
Retired	18 (45)	22 (55)	
Literacy *N* (%)			0.6
Primary or below	24 (27.3)	64 (72.7)	
Secondary education	24 (21.6)	87 (78.4)	
Post-secondary education	18 (26.1)	51 (73.9)	
Duration of diabetes years: mean, SD	9.3 (5.4)	6.6 (5.4)	0.0005
Diabetes in family member *N* (%)			
Yes	16 (25)	48 (75)	0.9
No	50 (24.3)	156 (75.7)	
Comorbid high BP *N* (%)			
Yes	31 (30.7)	70 (69.3)	0.07
No	35 (20.7)	134 (79.3)	
Knowledge of diabetes eye complications *N* (%)			0.003
Yes	47 (31.3)	133 (68.7)	
No	19 (28.8)	101 (84.2)	

For the 109 (40.4%) who had knowledge that diabetes causes complications, the complications that were of concern were losing a leg 34%, kidney failure 31.2%, stroke 22% and blindness 9%. Although 150 (55. 6%) knew that diabetes can affect the eye, 18.9% of the participants felt that there was no need for an eye examination and 42.6% would only go for an examination if they developed ocular symptoms.

## Discussion

The results indicated that both initiation and maintenance of annual fundoscopy is low. This may be due to the lack of systematic DR screening programmes in the country. Similar findings have been documented in other resource-poor settings (Table 1) [19, 20, 32–36]. The findings can be generalised to examination for DR in adult PLWD populations in Kenya since the study included any PLWD above 18 years in three geographical locations representing the rural-urban continuum within the diabetes belt. The lowest uptake was in Kirinyaga, suggesting that the macro environment in which PLWD live is a determinant of uptake [15]. Referral by the diabetic clinic for an eye examination, positive opinion on need for an eye examination and knowledge of diabetes eye complications are the modifiable factors that were positively associated with uptake of examination.

Sociodemographic attributes of patients were found to affect uptake of examination. The heterogeneity by county reflects geographic, social, cultural and/or economic influences. Rural populations are known to have low access to screening services [20]. This could be related (in part) to a rural-urban gap in awareness, resources or empowerment [37]. Paksin-Hall et al. [38] found income level, education level and health insurance status to be important determinants of annual dilated eye examinations, but these were not significant independent predictors in this study.

In previous studies, increasing age was a predictor for having an eye examination [39, 40]. In our study, the evidence for this association with strong for eye examination ever (*p* < 0.0001) and weak for an examination in the last year (*p* = 0.08). Although the effect size was small, the findings of an association are consistent with an increased likelihood of examination with age. Given that the risk of developing DR increases with age, older adults, more than any other age group, need to have regular eye examination, and as the population is aging, an expanding need for retinal examination in the country is predictable. Duration of diabetes is an important predictor for incidence and prevalence of DR, [40–42] so as more people live longer with diabetes, the need for an annual eye examination will increase.

Gender was not a predictor of uptake of examination, although there was very weak evidence that male gender was a predictor for referral (*p* = 0.09). A positive family history of diabetes was similarly not a predictor of uptake of examination, which suggests that barriers to access are not just at the individual level but also within households [15].

Hypertension in PLWD was a positive predictor of uptake of eye examination in this study, as also reported in another study [19]. Comorbidity is known to increase health care utilization in diabetes, [43] and hypertension is a common vascular comorbidity [11, 33, 35, 37, 40]. Uncontrolled hypertension is a risk factor for development of DR. There was weak evidence that PLWD with hypertension were more likely to be referred (*p* = 0.07) than normotensive PLWD, perhaps because the diabetes is considered more severe. This association strengthens the case for integration of eye care into non-communicable disease care.

There was very strong evidence that knowledge of any diabetes eye complication increases the uptake of examination (Table 3). Other studies have also found that knowledge is a predictor for uptake of screening [19, 20, 42]. However, in this study, only 9% listed blindness as a complication that they were concerned about. Another finding in this study is that PLWD need knowledge about the necessity and the frequency of eye examinations. Nearly half (42.6%) of the participants thought that DR screening should be symptom-led, which is a misconception that can lead to delay in getting an examination and treatment resulting in visual loss. Educational messages need to be tailored to an awareness of eye complications from diabetes and the need for diabetics to have the eyes examined once a year. This tallies with the finding that the most frequently reported suggestion for improvement given by PLWD was the need for more information/education. Thus, there exists a real opportunity for demand-driven health education.

Although there was strong evidence that knowledge of diabetes eye complications is a predictor of examination, there exists a gap between possessing this knowledge and the uptake of examination. About 56% of PLWD knew that diabetes causes eye complications, but only 25% of all PLWD had ever received an eye examination. Similarly, although approximately 25% were given a referral, only 13% had actually gone for the examination in the last year. This suggests that there are additional factors besides knowledge and referral that influence uptake.

**Fig. 1:**
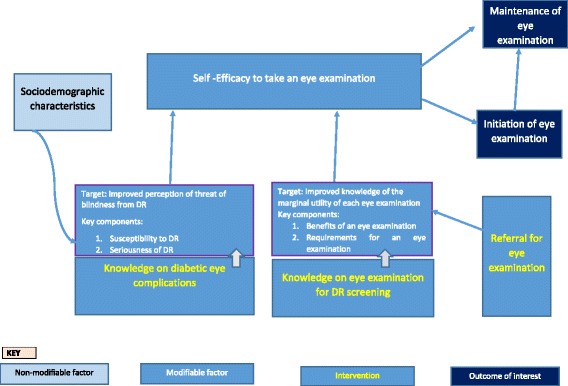
A conceptual model on how interventions to strengthen knowledge of PLWD, referral and self-efficacy can improve uptake of eye examination

The health belief model (HBM) is a widely used theoretical framework for understanding health behaviours within public health. Weiss et al. have previously shown that behavioural interventions can improve uptake of eye examination [44]. Taking the predictors found in this study into consideration, and using HBM as a theoretical framework, we conceptualise that self-efficacy is on the pathway between knowledge, referral and uptake of examination (Fig. 1). Research has shown that health behaviours such as taking an eye examination are associated with self-efficacy. In turn, self-efficacy can be increased in four ways: performance accomplishment, vicarious experience, verbal persuasion and psychological cues [45]. We postulate that interventions that increase knowledge, referral and self-efficacy can increase uptake of eye examination. Our conceptual model captures these different aspects (Fig. 1).

Only a quarter of PLWD had received a referral for DR screening. Similarly, in other studies in China and India, less than a half had been referred [19, 20], although in one study in India, over 60% had a referral [35]. We found that the strongest predictor for having an eye examination was referral from diabetes services. Participants already attend the diabetes clinic every 4 months because of the recommendation of the diabetes services. As there is no systematic DR screening programme, a referral to the eye clinic is a crucial bridge. These three visits a year are missed opportunities for referral for eye examination. Lack of a diabetes provider’s recommendation has been documented as a barrier in Germany [9] and Paraguay [42], diabetes services being gate keepers to other services required by PLWD. Written communication from the patient’s ophthalmologist to the primary care provider has also been found to increase adherence to future dilated eye examination [46]. Conversely, as entry to the eye clinic in Kenya does not actually require a formal referral note from diabetes services, an intervention that empowers patients for ‘self-referral’ might increase uptake of the examination.

There was strong evidence that older people with diabetes, those with longer duration of diabetes and those with a knowledge of diabetes eye complications were more likely to be referred. There was evidence that PLWD in Kirinyaga were less likely to be referred than those in Nakuru or Nairobi. Interventions to strengthen the use of clinical guidelines can ensure that all PLWD get a referral for an annual eye check.

### Limitations

This study has several limitations. First, as this is a cross-sectional study, a temporal relationship cannot be established between the predictor factors and the uptake of screening. In addition, the association between the variables is still subject to residual confounding by unmeasured variables such as distance from home to the eye clinic, medical conditions such as depression, disability and membership of diabetes support groups. Self-reported data was used and is prone to recall bias and social desirability bias. Under reporting of health behaviours, such as the duration since the participant had the last eye examination, may introduce information bias. This is a clinic-based study and did not include PLWD not attending diabetes services; however, we presume that they would have an even lower uptake of screening for DR.

## Conclusions

There is poor compliance with recommendations for annual eye examination among PLWD who have access to diabetes services. An intervention targeted at motivating adherence is essential. Such an intervention should empower PLWD to request/demand an eye examination and strengthen knowledge, referral and self-efficacy.

The opportunity to increase uptake of eye examination is also a valuable avenue for integrating diabetes care and eye care. Programmes to increase awareness regarding the importance of eye examinations can be combined with interventions to improve blood pressure monitoring and other aspects of diabetes management.

### Implications

Our study has demonstrated the low uptake of screening for DR by PLWD and described the attributes associated with uptake of eye examination. Low uptake has adverse effects at individual level and at the health system level because of the associated increased risk of blindness from DR. The low uptake highlights barriers in the link between diabetes services and eye care services. There is need to integrate screening for DR within the routine diabetes services and to implement interventions to increase uptake of screening.

### Future work

As the burden of diabetes grows over the next decade, there is a need to investigate the trend in uptake of annual eye examination and to examine sustainable interventions that can maximise increase uptake for eye examination. There is also need to investigate why there is a lack of attention to DR screening among diabetes clinicians and to evaluate the effect of providing them with clinical decision-making tools such DR guidelines.

## Abbreviations

CI: Confidence interval
DM: Diabetes mellitus
DR: Diabetic retinopathy
HBM: Health Belief Model
PLWD: Persons living with diabetes
SD: Standard deviation
STDR: Sight-threatening diabetic retinopathy
